# Impact of different food label formats on healthiness evaluation and food choice of consumers: a randomized-controlled study

**DOI:** 10.1186/1471-2458-9-184

**Published:** 2009-06-12

**Authors:** Ingrid Borgmeier, Joachim Westenhoefer

**Affiliations:** 1 Public Health Research Group, Department of Health Sciences, Faculty of Life Sciences, Hamburg University of Applied Sciences, Lohbruegger Kirchstrasse 65, 21033 Hamburg, Germany

## Abstract

**Background:**

Front of pack food labels or signpost labels are currently widely discussed as means to help consumers to make informed food choices. It is hoped that more informed food choices will result in an overall healthier diet. There is only limited evidence, as to which format of a food label is best understood by consumers, helps them best to differentiate between more or less healthy food and whether these changes in perceived healthiness result in changes of food choice.

**Methods:**

In a randomised experimental study in Hamburg/Germany 420 adult subjects were exposed to one of five experimental conditions: (1) a simple "healthy choice" tick, (2) a multiple traffic light label, (3) a monochrome Guideline Daily Amount (GDA) label, (4) a coloured GDA label and (5) a "no label" condition. In the first task they had to identify the healthier food items in 28 pair-wise comparisons of foods from different food groups. In the second task they were asked to select food portions from a range of foods to compose a one-day's consumption. Differences between means were analysed using ANOVAs.

**Results:**

Task I: Experimental conditions differed significantly in the number of correct decisions (p < 0.001). In the condition "no label" subjects had least correct decisions (20.2 ± 3.2), in the traffic light condition most correct decisions were made (24.8 ± 2.4). Task II: Envisaged daily food consumption did not differ significantly between the experimental conditions.

**Conclusion:**

Different food label formats differ in the understanding of consumers. The current study shows, that German adults profit most from the multiple traffic light labels. Perceived healthiness of foods is influenced by this label format most often. Nevertheless, such changes in perceived healthiness are unlikely to influence food choice and consumption. Attempts to establish the informed consumer with the hope that informed choices will be healthier choices are unlikely to change consumer behaviour and will not result in the desired contribution to the prevention of obesity and diet related diseases.

## Background

Overweight and obesity are an increasing problem. Worldwide more than 1,6 billion people (age 15+) are overweight, and approximately 400 million adults are obese [1]. Germany is one of the countries with the highest prevalence of overweight among adults (BMI >25) in the European Union [2]. The German Child and Adolescent Health Survey (KiGGS) has demonstrated that overall 15% of children and adolescents between the ages of 3 and 17 are overweight, and 6.3% suffer from obesity [3,4]. This represents a 50% increase in the prevalence of overweight and more than 100% increase in the prevalence of obesity as compared to the BMI reference data from 1985–1999 [5]. Recently, the German National Nutrition Survey II (NVS II) [6] a representative survey on the nutrient and energy intake of 14–80-year-olds determined that one in five Germans is obese (BMI ≥ 30). Analyses on current food consumption, lifestyle and eating behaviour demonstrated that 36% men and 31% women exceeded the guidelines for daily energy intake for median physical activity. Of even more concern are the results for the daily fat intake: 80% men and 76% women exceed the daily fat intake recommendations (30% of total energy intake) [7]. In almost the same manner eating habits have changed worldwide leading to an increased consumption of pre-packed food generally containing high levels of sugar, fat, saturated fatty acids, trans-fatty acids and sodium [8]. To prevent nutrition related diseases the WHO has recommended to reduce these nutrients in order to improve the nutritional value [9]. Hence consumer interests in health and diet issues have increased and consequently nutrition labelling has received considerable attention. Interpretational aids can help consumers to appraise the nutrient contribution of specific foods to the overall diet enabling informed consumer choices, leading to the consumption and consequently to the production of healthier products [10-12]. However, research on nutrition information on packed foods showed that the given information is often misinterpreted, confusing and inappropriate for estimating an individual product's contribution to the overall diet [11,13,14]. Adding some kind of benchmark either numerical or non-numerical seems to help [11,12]. Label use is affected by education, gender, age and time pressure, i.e., consumers search for nutrition-related information as long as the costs (time and/or price) will not outweigh the benefits [14].

In addition to the customary back-of-pack labelling formats, several food manufactures are using signposts on the front of the packages to help consumers interpret the nutritional information. Signposts could probably change eating patterns by informing and supporting consumers to make healthy choices. However, the effectiveness of such labelling depends on the organisation and presentation of the information, implying the importance of regulatory issues [10].

In Europe nutrition labelling is compulsory if a nutrition claim is made. Depending on the nutrition claim either the contents of energy, protein, carbohydrate and fat (so called "big 4") or these four plus sugars, saturated fat, fibre and sodium.(so called "big 8) have to be detailed [15,16]. Due to the lack of binding agreements, there is no European standard guideline on front-of-pack or signpost labelling regulations. Nevertheless, several signpost labels have already been developed although their effect on consumers is highly controversial.

Examples of detailed labels are the 'Guideline Daily Amounts' (GDA) showing the total amount of energy and nutrients as a percentage of what a typical healthy adult should be eating daily on the basis of a 2000 kcal diet. The traffic light-labels (simple or multiple) give information on the level (i.e. high, medium or low) of individual nutrients in the product using the colour coding red, amber or green. The colour coded GDA combines the two previous label systems. Studies on behalf of the UK Food Standard Agency showed that the colour coded labels like the multiple traffic-light and coloured GDA formats are most accepted and well understood by consumers [17-19].

Results from a UK market study on food labels indicated a high awareness of both, GDA and traffic light label systems. In terms of understanding, the GDA concept is good. The understanding of the traffic light concept seems to be characterised by some exaggeration of the meaning of the colours [20].

More or less simple labels are the health logos "pick the tick" in Australia and New Zealand, a tick symbol for approved foods low in total fat, saturated fat, added sugar and sodium, and the "smart spot" in the USA for products meeting similar nutrition criteria [21-23]. In the Netherlands criteria for "the healthy choice" logo, a single tick on the front of the package are derived from WHO standards of trans fat, saturated fat, sugar and sodium [24].

Research on consumers' use of various formats of nutrition labels has been mainly undertaken by consumer associations, major retailers and the Food Standards Agency, focusing on the liking of various signpost labels [25]. In general, most customers like the idea of front-of-pack signposting as a shopping aid. In addition most consumers state that they understand the information provided by these labels, which may be characterised as perceived or subjective understanding. However, there is virtually no insight into how labelling information will be used in a real-world shopping situation and how it will affect consumers' dietary patterns. Research to evaluate differences in the consumers' objective (actual) and subjective (perceived) understanding of various label formats is still to come [25]. Only a few studies investigated the effect of different formats on behavioural change [20,21,26]. This makes it difficult to identify the everyday use of (different) nutrition labels and their influence on diet quality.

### Objectives

The objective of this research was to investigate which signpost food label format enables consumers best to differentiate healthier products from less healthy ones and to examine the impact of these food labels on the food choice and quality of diet.

## Methods

### Study design and experimental conditions

**Figure 1:**
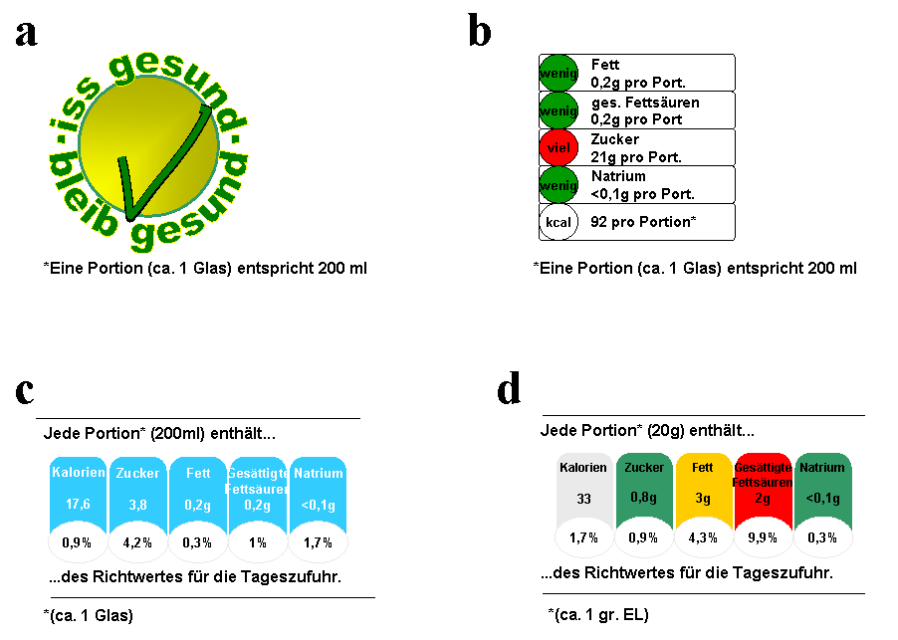
**The nutrition labelling formats (a) simple tick; (b) traffic light format; (c) monochrome Guideline Daily Amount (GDA); (d) colour-coded GDA (CGDA)**. Translation Figure 1. 1.a) "eat healthy – stay healthy". Portion size (approx. 1 glass) is 200 ml. a-d) one portion (approx. 1 glass) is 200 ml. b-d) wenig: low; mittel: medium; viel: high; Kalorien: calories; Fett: fat; gesättigte Fettsäuren: saturated fatty acids; Zucker: sugar; Natrium: sodium. 1.c) each portion (200 ml/approx. 1 glass) contains xx% of your guideline daily amount. 1.d) each portion (20 gm/approx. 1 Tablespoon) contains xx% of your guideline daily amount.

A randomized experimental study was conducted using 5 experimental conditions representing different label formats. Four different label formats (signposts) were examined (see figure 1). The study protocol has been approved by the ethical committee of the Life Sciences Faculty of the Hamburg University of Applied Sciences.

### Materials/food cards

Foods from different food categories were photographed, and the photos printed in postcard size. All food pictures were presented unbranded to the participants. In the experimental condition with the food label signpost, this was located below the product as was the information about the portion size (see figure 1). In the experimental condition without the signpost only the portion size was presented below the picture. If nutrition facts were available for the reference food (e.g. front-of-pack nutrition labelling, website information, requested information via telephone or email) this data was taken and adapted to the different labels. If product information was not available the ingredients were calculated in accordance with the technical guidelines using nutrient content tables or computerized nutrition content programmes for similar products [27-30].

### Food Label formats

1. A simple healthier choice tick (tick), similar to the Dutch "Ik kies bewust", the American "smart choices" or the "pick the tick" logo of New Zealand's National Heart foundation was given, if the food qualified for the green colour, for each, sugar, fat, saturated fat and sodium in the multiple traffic light system. For foods which did not meet the criteria no logo, but only portion size was shown. Thus the "tick" label represents a combined evaluation of the food's nutrient content, whereas the other label formats give separate evaluations for the different nutrients.

2. In the multiple traffic light label system (t-l) the corresponding colour for the content of fat, saturated fat, sugar and sodium were given. Green, amber and red represent low, moderate and high contents of the nutrients in the food product, respectively. The colours are based on concentration in grams per 100 g or per 100 ml and the criteria of the UK Food Standards Agency were applied to assign the colour codes [31]. In addition calories were shown in neutral colour (white/grey).

3. The Guideline Daily Amount (GDA) labels showed the amount of fat, saturated fat and sugar and sodium in grams as well as the kilocalories per portion and the percentage of each of these ingredients according to the "Guideline Daily Amount for women" [32].

4. The colour Coded GDA labels (CGDA) were similar to the GDA labels. In addition to the specified amounts of nutrients a green, amber or red colour symbol analogue to the traffic light criteria was given.

5. As an additional experimental condition we used "no label" where the foods were shown only with portion size information without any additional label.

For all five experimental conditions foods were presented with a specification of portion size in gram or millilitre and an additional explanation (e.g. "portion size is 200 ml, this equates approximately 1 glass").

After answering some questions on demographic variables, body weight and height, each subject received a short, standardised description of the label format of his or her experimental condition and its meaning and after that completed two tasks: 1. Pair-wise comparison of foods and 2. virtual grocery.

#### Task 1: Pair-wise comparison of foods

Participants were shown pictures (as described above) of 28 food pairs from the following food groups: drinks (3 pairs), fat/oils/sauces (4 pairs), meat/fish/sausages (6 pairs), dairy products (8 pairs), bread/cereals/grain products (3 pairs), fruits/vegetables (1 pair) and candies/snacks (3 pairs). The selection of the food pairs was made with the attempt to produce a representative sample of foods from the different food categories commonly eaten. Dieticians with experience in applied nutritional counselling were interviewed to find the appropriate food items. These food items were selected only if a healthy alternative was available and commonly eaten. Photographed foods were shown on cards with portion size information and food label corresponding to the experimental condition. Each food pair consisted of a healthier and less healthy variant. The healthier variant had less fat, saturated fat, sugar, sodium or energy than its counterpart. In case of bread for e.g. the healthier variant was 'whole grain bread' and 'whole grain toast' (see Additional file 1).

Participants were informed that the presented food pairs were labelled with a signpost or, in case of "no label", with no nutrition label. It was explained, that the purpose of the study was to investigate whether food labels help to identify the "healthier variant, particularly with regard to a healthy body weight" of different foods. Then successively each pair of food cards was presented and participants were asked to identify the healthier food in each pair.

#### Task 2: Virtual grocery: Envisaged daily food consumption

For the virtual grocery task a shopping situation was simulated. 78 food pictures (including the 56 from task one) were placed in front of the participants, arranged in different food groups (identical to the categories in task one). In the same manner as for task 1 each food picture was labelled with the portion size and the signpost/or not of the experimental condition to which the participant was allocated.

The participants were asked to simulate a shopping situation, in which they select all foods and drinks, they would like to consume on the next day (e.g. for breakfast, lunch, dinner, snack). They were told that they could only select from the presented foods and were asked to specify how many portions they will buy for the envisaged consumption for the next day.

The presented foods were chosen in order to enable the participants to compose a day's consumption pattern that would comply with current dietary guidelines and recommendations [7]. The 78 foods could only represent a small variety of food items usually available in supermarkets. Therefore, the participants were asked to imagine being in a small "grocery around the corner" with a limited range of products. In addition they were told that the products' flavours shown on the photos in particular for yoghurt, jam or fruits could be substituted by other liked flavours.

In order to assess the possible impact of food labels on usual food choice the subjects were not explicitly prompted to make healthy food choices. However, since the virtual grocery was the second task after having identified the healthier food variants in task one, and the 56 food pictures from task one were used again in this task, there might have been an implicit inclination towards healthy food choices.

### Subject recruitment and randomisation

A total of 30 interviewers investigated 14 subjects each. Each interviewer was asked to recruit a convenience sample of 7 female and 7 male subjects aged between 18 and 80 years. Subjects with training or work experience in the nutrition sector (e.g. dieticians, nutritionists, physicians, fellow dietetic students, etc.) were excluded.

Each of the 30 interviewers was assigned to investigate 2 of the 5 experimental conditions. Since there were 10 possible pair wise combinations of the 5 conditions (e.g. no label – simple tick, no label – traffic light ...), 3 interviewers were assigned to each of 10 pair wise combinations using a random number generator. Each interviewer received a list – again randomly generated – that detailed the sequence in which his or her 14 interview subjects were assigned to the experimental conditions. Thus, each of the 420 subjects was assigned to one experimental condition, i.e. one label format randomly. This procedure yielded 84 subjects within each of the five experimental groups.

### Statistical analysis

Statistical analyses were done using SPSS Version 16 for Windows. Descriptive statistics are shown as mean ± standard deviation unless noted otherwise. From self-reported body weight and height Body Mass Index (BMI) was computed as BMI = kg/m^2^. For task 2 (virtual grocery) the total energy and nutrient intake as well as the energy density of the envisaged food consumption was computed using the nutrition information for the sample foods. Group differences in both tasks were examined using chi square tests for categorical variables and one way ANOVAs for continuous variables. To examine significant differences between means in more detail, post-hoc t-tests were computed after significant ANOVA results. Since these post-hoc test had exploratory character we abstained from adjusting significance levels for multiple testing.

In order to investigate whether sex, educational level and body weight status had an additional influence two-way ANOVAs were computed using experimental condition as the first factor and sex, educational level or weight group as the second factor. In the analysis of educational level 2 subjects were excluded who did not report an appropriate answer. For the analysis of weight status two groups were built: normal weight subjects with a BMI < 25 (including some underweight subjects) and overweight subjects with a BMI ≥ 25 (including some obese subjects). The significance level for all tests was set to p < 0.05.

## Results

### Participants

In total, 420 participants took part in this study. 84 subjects participated in each of the 5 experimental groups. The sample characteristics are described in table 1. The experimental groups did not differ significantly with regard to sex distribution (CHI-square = 1.8; df = 4; p = 0.769), average age (F = 0.29; df = 4/415; p = 0.886) average BMI (F = 1.02; df = 4/415; p = 0.396) and educational level (CHI-square = 16.0; df = 12; p = 0.19). The BMI ranged from 17.1 to 43.3. N = 278 subjects (66.2% of the sample) had a BMI below 25, and n = 142 (33.8%) had a BMI of 25 or more.

**Table 1:** Socio-demographic characteristics and BMI of the study participants

	No label	Tick label	Traffic light	GDA	CGDA	Total	Sig.
Number (%)	84 (20%)	84 (20%)	84 (20%)	84 (20%)	84 (20%)	420 (100%)	
Sex							n. s.
- Female (%)	49 (21.8%)	44 (19.6%)	44 (19.6%)	47 (20.9%)	41 (18.2%)	225 (53.6%)	
- Male (%)	35 (17.9%)	40 (20.5%)	40 (20.5%)	37 (19.0%)	43 (18.2%)	195 (46.4%)	
Age (years)	36 ± 15	36 ± 13	35 ± 13	36 ± 14	38 ± 16	36 ± 14	n. s.
BMI (kg/m^2^)	23.3 ± 3.1	24.1 ± 3.5	24.1 ± 3.5	23.8 ± 4.0	24.4 ± 4.1	24.0 ± 3.7	n. s.
Education							n. s.
- Low^1 ^(%)	3 (7.5%)	12 (30.0%)	7 (17.5%)	7 (17.5%)	11 (27.5%)	40 (9.5%)	
- Middle^2 ^(%)	23 (16.5%)	29 (20.9%)	24 (17.3%)	32 (23.0%)	31 (22.3%)	139 (31.1%)	
- High^3 ^(%)	58 (24.3%)	42 (17.6%)	52 (21.8%)	45 (18.8%)	42 (17.6%)	239 (56.9%)	
- Other (%)	-	1 (50%)	1 (50%)	-	-	2 (0.5%)	

^1^low = no school degree, secondary general school (usually 9 years)

^2^middle = intermediate secondary schools or special upper secondary school (10 – 12 years)

^3^high = general higher education or university degree (13 years or more)

n. s.: not significant

### Task one: Pair wise Comparison of foods

Additional file 1 shows the percentage of subjects within each experimental condition which correctly identified the healthier alternative in the pair wise comparison of foods. Significant differences between the percentages of correct healthy choices were found for 22 of the 28 food pairs. In 14 comparisons with significant differences the traffic light label yielded the highest percentage of correct choices. In addition, the traffic light label yielded the highest percentage of correct choices in 5 comparisons without significant differences. The highest percentage of correct choices was shared with GDA in two food pair comparisons and with coloured GDA in two other comparisons. In 2 comparisons the highest percentage of correct choices was associated with the GDA label, plus 2 comparisons where the highest percentage of correct choices was shared with the traffic light label. The coloured GDA label shared the highest percentage of correct choices in two pairs with the traffic light label. The simple "tick" had the highest percentage of correct choices in four comparisons (including one without significant differences); "no label" was only in one pair wise comparison associated with the highest percentage of correct choices.

The average number of correct choices for each subject differed significantly between the experimental conditions and hence the different signpost formats (F = 28.7; df = 4/415; p < 0.001) (see table 2). The traffic light label yielded the highest average of 24.8 correct choices between the 28 pairs. The "no label" condition was associated with the lowest average of correct choices (20.2 of 28 pairs). To examine these differences in more detail post-hoc t-tests between each pair of label formats were computed. The average number of correct choices did not differ significantly between the GDA and coloured GDA format. All other comparisons yielded significant differences in the number of correct choices between the different label conditions (p < 0.01).

**Table 2:** Mean number of correct healthier choices (± SD) in the pair wise comparison task (Task 1)

	No label	Tick label	Traffic light	GDA	CGDA	Total
Total (1)	20.2 ± 3.2	21.5 ± 2.7	24.8 ± 2.4	22.8 ± 3.2 (a)	23.1 ± 3.1 (a)	22.5 ± 3.3
Sex (2)						
- Female	20.7 ± 3.3	22.3 ± 2.0	24.7 ± 2.5	23.2 ± 2.7	23.4 ± 2.3	22.8 ± 2.9
- Male	19.5 ± 2.9	20.8 ± 3.1	24.8 ± 2.3	22.3 ± 3.7	22.8 ± 3.7	22.1 ± 3.6
Education (3)						
- Low	19.0 ± 1.7	21.4 ± 2.9	23.6 ± 1.4	23.3 ± 2.4	21.8 ± 5.0	22.1 ± 3.4
- Middle	20.4 ± 3.3	22.2 ± 2.2	24.5 ± 2.9	22.1 ± 4.0	23.2 ± 2.9	22.5 ± 3.4
- High	20.2 ± 3.2	21.3 ± 2.8	25.1 ± 2.2	23.2 ± 2.5	23.4 ± 2.6	22.6 ± 3.2
Weight group (4)						
- BMI < 25	20.4 ± 3.4	21.9 ± 2.6	25.0 ± 2.0	23.0 ± 3.0	23.6 ± 2.9	22.8 ± 3.2
- BMI ≥ 25	19.9 ± 2.6	21.0 ± 2.8	24.2 ± 3.0	22.4 ± 3.5	22.2 ± 3.4	21.9 ± 3.4

(1) A one-way ANOVA of experimental conditions (label format) yielded a significant main effect (p < 0.001)

(a) Means were not significantly different in post-hoc t-tests

(2) A two-way ANOVA of label format x sex yielded significant main effects for label format (p < 0.001) and sex (p < 0.01)

(3) A two-way ANOVA of label format x educational level yielded only a significant main effect for label format (p < 0.001).

(4) A two-way ANOVA of label format x weight group yielded significant main effects for label format (p < 0.001) and weight group (p < 0.01)

To explore possible influences of gender, educational status and weight status additional two-way ANOVAs were computed. The two-way ANOVA using label format and sex as independent factors yielded significant main effects for both label format (F = 30.0; df = 4/410; p < 0.001) and sex (F = 7.9; df = 1/410; p < 0.01). The interaction between the two factors was not statistically significant (F = 7.6; df = 4/410) indicating that the influence of gender did not differ between the experimental conditions. Inspection of the means (Table 2) shows that women had a slightly higher average number of correct choices. The two-way ANOVA using label format and educational level as independent factors yielded only one significant main effect for label format (F = 11.0; df = 4/403; p < 0.001). Neither the main effect of education (F 0 1.1; df = 2/403) nor the interaction (F = 0.9; df = 8/403) attained significance. The two-way ANOVA using label format and weight group as independent factors yielded significant main effects of label format (F = 24.2; df = 4/410; p < 0.001) and weight group (F = 8.1; df = 1/410; p < 0.01). The interaction between these factors was not significant (F = 0.2; df = 4/410) indicating that weight status did not affect the number of correct decision differentially between the experimental conditions. An inspection of the means (Table 2) showed that overweight subjects had a slightly lower number of correct decisions compared to normal weight subjects.

### Task 2: Envisaged daily food consumption (Virtual grocery)

The envisaged daily consumption of energy and nutrients which was computed from the food choices in the virtual grocery task. The means and standard deviations are described in Additional file 2 for the 5 experimental groups. A comparison with the corresponding values from the German national nutrition survey II [33] is provided in Additional file 3. In all experimental groups the average daily intake for fat, saturated fat, sugar and sodium was above the recommendations for daily consumption (Additional file 2). One way ANOVAs were used to examine differences between the experimental conditions. Intakes of energy, nutrients in gram and nutrients as percentage of energy intake did not differ significantly between the examined label formats.

To analyse possible interactions between subject characteristics and label format, additional two-way ANOVAs were computed using label format as one factor, and sex, educational level and weight status as the second factor. The analysis using sex as the second factor yielded significant main effects for sex for all outcome parameters except energy-percent of protein. Men had significantly higher envisaged consumptions of energy, grams fat, grams saturated fat, grams protein, grams sugar, grams carbohydrates, grams sodium, energy percent from fat, energy percent from saturated fat, lower energy percent from carbohydrates, and a higher energy density of chosen foods. Neither the main effect of food labels nor the interaction between sex and food labels attained significance, indicating that these sex differences were similarly present for each label format.

Using educational level as a second factor, the two-way ANOVA yielded a significant interaction between label format and education for sodium intake (in grams) (F = 2.2; df = 8/403; p < 0.05) and protein (in grams) (F = 2.4; df = 8/403; p < 0.05). All other effects did not reach statistical significance. Higher sodium intake was associated with higher education in the traffic light and coloured GDA condition, but with lower educational level in the simple tick condition (means are not presented in this paper). No systematic variation was found for the two other labels. For proteins, increasing educational level was associated with lower intake in the simple tick condition, with higher intake in the traffic light condition and with no systematic relationship in the other label conditions.

In the analysis using weight status as a second factor only the two-way ANOVA on energy percent from carbohydrates yielded significant main effects of experimental condition (F = 2.8; df = 4/410; p < 0.05) and BMI group (F = 5.0; df = 1/410; p < 0.05). An inspection of the means (not presented in detail in this paper) showed that normal weight subjects had higher energy percentages from carbohydrates than overweight subjects. The energy percent from carbohydrates was lowest in the CGDA-condition and highest in the "no label" condition (see Additional file 2).

## Discussion and conclusion

Aim of the present paper was to investigate whether different formats of signpost food labels help consumers to differentiate between healthy foods, healthy particularly with respect to body weight, and less healthy foods, and whether they are likely to have an impact on consumers' food choices.

So far some research has been conducted on the subjective understanding of nutrition information. Studies show, that consumers think they do understand the nutrition information on food packages correctly which has been called subjective understanding [25]. However, less work has been done to assess the actual, objective understanding of such information. In our study we assessed the objective understanding of nutrition information given in different signpost food label formats by testing whether subjects were actually able to identify the healthier food variants.

Results from our study clearly indicate that signpost labels help to identify healthier foods better than un-labelled food. We also found differences in the efficacy of the different label formats to support the correct decision about healthier food variants. In our study, clearly, the multiple traffic light system showed the best performance. For most of the pair-wise comparisons the traffic light format showed the highest percentage of correct choices, and also the overall number of correct decisions was highest in this format. However, the differences between the different label formats were only moderate. Without any signpost label, the study participants correctly identified the healthier food in 20.2 of 28 pair-wise comparisons, while in the multiple traffic light label system correct choices were made in 24.8 of 28 cases. The simple "healthy choice" tick, the monochrome GDA label and the coloured GDA label resulted in an average number of correct decisions between no label and multiple traffic light label. Interestingly enough, adding traffic light colours to the monochrome GDA showed only a modest, statistically not significant increase in the number of correct choices. The results point partially to foods or food groups where food labels may be of particular value. In our dataset without food labels only a minority of subjects was able to identify milk chocolate as the healthier alternative in terms of fat, saturated fat and sugar. And even with food labels a large portion of subjects comes to a wrong decision regarding the "healthy variant particularly with regard to a healthy body weight". Presumably people use in their health evaluations knowledge from the media which portrayed dark chocolate as healthy due to its health protecting ingredients as e.g. flavonoids. Such information seems to overwrite other information even if people are prompted to consider body weight.

In addition we did not find that the various signpost formats affected the different subject groups differently. In general, women in comparison to men and normal weight subjects in comparison to overweight subjects had a higher number of correct decisions, but since this was the case for all experimental conditions it was independent from the label format. Interestingly enough, we also failed to find an influence of education status on the number of correct decisions.

These outcomes are consistent with research from the Food Standards Agency, London (FSA) where consumers could imagine using food label information, especially when the information is colour-coded, and in cases where there is a range of product alternatives in the same product category, the items may differ in their "colour range" [25,34]. A recent study, which is not fully published yet, revealed a good understanding of the nutrition labelling schemes used in the UK. Most consumers were able to rank food products correctly in terms of healthiness regardless of the labelling system [20,35]. This corresponds to the only modest difference in the number of correct decisions of healthier food choices among the different food label formats in our study.

If nutrition information given in the form of signpost food labels has an impact on the perceived healthiness of foods, it is an independent question whether the perceived healthiness is likely to ultimately influence food choice and food intake.

A recent pre-published study shows that only one in four shoppers actually looks for nutrition information on food packages in supermarkets [20]. It is obvious that most consumers read nutrition information when exposed to it accidentally rather than seeking for it deliberately [25]. In addition, label use is negatively linked to time pressure [25]. On average European consumers spend between 25 seconds (UK) and 47 seconds (Hungary) per product bought in a supermarket [35]. Consequently several situational, behavioural and attitudinal factors like income, time or household size have an effect on the use of on-pack nutrition information and therefore influence on the food choices [14]. Furthermore, nutrition labelling is only one of several information sources available to consumers. Media, advertising and promotion activities can also affect consumers' choices [10,36]. This may prompt nutritionists to consider the limitations of nutrition labels for health promotion [21].

Former research has shown that the self-reported use of food labels is associated with lower fat intake [37-39] and higher fruit and vegetable intake [37]. However, the causal nature of this association is not quite clear. Indeed, results from a study by Lin et al. [40] suggests that lower intake of fat, saturated fats and cholesterol will increase the probability of searching for information about these nutrients. In line with such considerations is the finding that prior nutritional knowledge predicts the use of food labels [41].

A recently published study found that subjects had the intention to change their future food choice when they were exposed to signpost labels of food products they usually eat and of food products that represented healthier variants of comparable food [26]. Subjects reported an intended increase of consumption frequency of less than one portion every two weeks of the healthier food variants. In addition they reported an intended decrease of consumption frequency of one portion per week of the usually eaten food for which they had been shown a healthier variant. There was no significant difference between different label formats.

In our study we asked our subjects to select foods they would like to eat during one day from a "virtual grocery". The resulting envisaged one day's food consumption was quite realistic, even though the total energy intake was slightly higher than the average energy intake in Germany, particularly in men [33]. In line with the results of Feunekes et al. [26] we could not find an influence of food label formats on any parameter of food consumption: Neither energy intake nor any of the examined nutrients or energy density of total envisaged food intake varied between the different experimental conditions. This pattern of results was maintained even if subject characteristics like gender, educational level or weight status were taken into account. For most parameters we found no statistical interaction of label format and subject characteristics indicating that the effect of label format was the same within each subject group. The only exceptions were sodium and protein intake in relation to educational level. However these variations were neither large nor systematic. These few significant interactions could be attributed to the multiple tests in this explanatory analysis.

Hence, results from the second task of our study show that even if a food's perceived healthiness is influenced by using signpost labels this is unlikely to have any major impact on the actual food choice.

This is in line with results from Grunert and Wills' review [25]: when buying intentions for products with or without signposts (health logos, traffic light, GDA-based systems) were measured, the use of labels would not prevent consumers from eating products which they like for their taste [25].

Despite the finding that food labels have no major impact on individual food choices, one effect of nutrition labelling – in particular with prominent symbols like traffic lights – could be the production of more healthy food products as manufacturers might wish to make their products more attractive with more favourable signposts [10]. This is endorsed by observations from Sainsbury's and Tesco which seem to indicate that, after introduction of their signposting system (the Wheel of Health, a colour-coded GDA system, for Sainsbury's and a not colour-coded GDA system for Tesco), sales of some healthier products went up whereas sales of comparable products with less favourable nutrient information went down [25]. Such changes would imply that signposting does change individual shopping behaviour. However, to our knowledge, the methods used and conclusions drawn from the Sainsbury's and Tesco findings have not been scientifically reported and can therefore be given only little credence.

Our study has some limitations which have to be discussed before drawing conclusions. First of all, our study sample was a convenience sample of adults recruited in the area of Hamburg (Germany). Thus our sample can not be considered as representative of the general population. This is particularly true, since we had a considerably higher number of subjects with high educational level than would be expected in a representative sample. We did not test or investigate nutrition knowledge of our subjects. Thus we can not exclude that a particular level of nutrition knowledge in our sample has contributed to our findings. In addition, owing to the fact that the interviewers recruited the study participants themselves, the potential for interviewer bias might be higher as the interviewers may have been more prone to recruit specific socio-demographic types, which could have influenced the results. However, given this limitation, analyses taking into account the different levels of education did not find any hints that educational level differentially affected the understanding of food labels, the perceived healthiness of foods resulting from the understanding of the food labels or the intended/envisaged consumption of food labelled in different formats. Nevertheless, increasing the sample size or including a substantially higher number of subjects with low educational status might increase the power to detect differences related to education which we missed with our convenience sample. In addition, our findings may not be valid for consumers in other countries, and we can not extrapolate from our findings in adults the possible impact food labels could have in children and adolescents.

A second limitation stems from the fact that we could only use a limited number of food items in our study. However, in investigating the understanding of food labels and the resulting perceived healthiness of foods we used 28 pairs of foods from different food groups which is considerably more than has been used in other studies [26,35]. In the second task of selecting food items for envisaged one-day consumption in a virtual grocery again we used only 78 foods, which is a very small number compared to the thousands of different products available in real supermarkets. Nevertheless, our subjects did not complain about this limitation and the resulting envisaged intakes were quite equivalent to average German consumption patterns with a slightly elevated energy intake in our study subjects, particularly in men. In addition to the limited number of foods, some bias might result from the fact that within the different food groups, different number of foods or food pairs were selected for the experimental procedure. The results of the pair-wise comparison task indicated that different labelling systems favoured different products. Thus the results may be biased by the over-representation of some product groups, e.g. we used 8 dairy product pairs in which the traffic light system most often produced the highest percentage of correct responses, but only three grain/cereal comparison in which the simple "tick" logo yielded the highest percentage of correct responses. Therefore a different composition of the number of foods within each food group may yield different results.

Of course, a third important limitation is that we examined only virtual, intended or envisaged food consumption and not real shopping or consumption behaviour. The selection of foods in this task for the envisaged consumption during one day, of course, is a rather artificial experimental situation. Thus, in real shopping situations a number of different factors and processes may influence shopping decisions and food choice. For example, promotion activities and discount offers may influence the shopping behaviour as well as shopping habits in a super-market that is familiar to the subject. In addition, in real life people eventually eat what they have bought, in this virtual task they were aware that their shopping choice was without real consequences. These differences may have biased our results. However, since we were interested in examining the influence of different food label formats on food choice there is little reason to believe that the labels would result in differences in real behaviour whereas they did not result in differences in our virtual, experimental situation. In addition, the experimental situation and the limited number of foods may contribute to the lack of differences in the virtual grocery task. The pair-wise comparison task showed that, overall there were only modest differences between the different label formats. Given these small differences it may be suspected that subjects would select a similar diet if they were asked to present a healthy food choice to the interviewer as might be implied by the experimental context. Thus, we can not exclude that a wider selection of foods might yield a better chance for the different labels format to produce differences in food choice.

A forth limitation lies in the criteria which we used for assigning the "tick" label. The "tick" label was only given, when all of the criteria to award the "green" colour for fat, saturated fat, sugar and sodium were fulfilled. This may represent a rather restrictive approach. Therefore results may be different if other approaches or criteria are used.

A fifth limitation is related to our conclusions regarding different body weight groups. We calculated BMI from self-reported weight and height which is known to be inaccurate and have restricted validity. Thus some of the subjects might have been misclassified as normal weight or overweight, and we can not exclude that results from weight related analyses would be slightly different if we had actually measured weight and height. However, all other inferences should not be influenced by this limitation.

Keeping in view of the limitations mentioned above, the results from our study would justify the following conclusions: (1) Signpost food labels may influence the perceived healthiness of food products by the consumer. (2) Different label formats differ in how effectively they help the consumer to identify the more healthy food when comparing different foods from the same food category. In our study, clearly the multiple traffic light format helped consumers most, to identify the healthier variant of foods. However, currently in real life, the German consumers are only exposed to the GDA food labels which are voluntarily printed on the products. Therefore, it is not clear whether the understanding of food labels will change when one or the other label format comes into wider use and becomes more familiar to consumers. In addition, for the sample foods of our study the decision which variant of a food was more or less healthy was unambiguous because the differences between the variants were only for one nutrient or for multiple nutrients in the same direction. It remains totally unclear how consumers would evaluate the healthiness of foods in a comparison where one food has for example a high sugar and low fat content whereas the other has a high fat, but low sugar content. (3) Despite the fact that food labels may influence the perceived healthiness of foods by the consumers, this is unlikely to have a major impact on food choice and consumption. Thus, there is little reason to assume that signpost food labels will be an effective instrument in the prevention of overweight and diet related diseases.

In our study, we were not able to address a number of issues and questions which should be addressed by future research. Children and adolescents are an important target group for the prevention of overweight and diet related diseases. Which type of food labels helps them best to differentiate between more or less healthy foods? Children and adolescents with migrant background have a particularly high prevalence of overweight and obesity in Germany [4]. Are there cultural differences in the understanding and use of food labels in groups with migrant and non-migrant background? Although we did not find differences in the understanding und use of food labels between different educational levels in our study, it may be useful to address socio-economic status as a primary variable in specifically designed studies. We also did not include motivational or attitudinal variables in our study. Future studies should examine whether subjects with different attitudes and/or motivations differ in their understanding of and profit from the use of different food labels. In addition, systematically examining, how other factors, particularly attitudinal and motivational variables that influence food choice, may be changed in order to achieve healthier food choices in different target population groups, may yield valuable information.

## Competing interests

JW has been consulting Mars Deutschland GmbH with regard to snacking behaviour of children.

IB has no competing interests.

## Authors' contributions

Both authors contributed similarly to the design of the study, analysis and interpretation of the results, and to the preparation, revision and final approval of the manuscript. In addition, IB has been primarily responsible for conducting of the study. All authors have read and approved the final manuscript.

## Authors' information

IB graduated in Public Health (Diplom-Gesundheitswirtin). She works as a research fellow at the Public Health Research Group of the Hamburg University of Applied Sciences.

JW is Professor of Nutritional Psychology and Health Psychology, Head of Public Health Research Group and Head of the Department Health Sciences at the Hamburg University of Applied Sciences, and Secretary of the German Society for the Study of Obesity.

## Pre-publication history

The pre-publication history for this paper can be accessed here:



## Supplementary Material

Additional file 1 **Percentage of correct choices in the pair-wise comparison task 1**. Food pairs used in the pair wise comparison task 1 (the less healthy food in brackets) and percentage of correct choices within each experimental condition (signpost format). The highest percentages are printed bold. Click here for file

Additional file 2 **Envisaged daily consumption of energy and nutrients (gram and energy percent) in the virtual grocery task (task 2)**. The data provided represent the mean ± standard deviation of the envisaged daily consumption of energy and nutrients in the virtual grocery task. Click here for file

Additional file 3 **Total envisaged daily consumption compared with the results of the German National Nutrition Survey II (NVS II)**. The data provided represent the mean ± standard deviation and the median of the envisaged daily consumption of energy and nutrients from the total sample (all experimental conditions combined) in the virtual grocery task and a comparison with the mean and median of intakes in the German National Nutrition Survey II (NVS II) [33]. Click here for file

## References

[B1] World Health Organization (2006). Obesity and overweight: What are Overweight and obesity? Fact sheet N°311.

[B2] International Association for the Study of Obesity IASO Adult overweight and obesity in the European Union (EU25). Overweight in Children in the European Union. http://www.iotf.org/documents/Europeandatatable_000.pdf.

[B3] Altgeld T, Geene R, Glaeske G, Kolip P, Rosenbrock R, Trojan A (2006). Prävention und Gesundheitsförderung – Ein Programm für eine bessere Sozial- und Gesundheitspolitik.

[B4] Kurth BM, Schaffrath Rosario A (2007). Die Verbreitung von Übergewicht und Adipositas bei Kindern und Jugendlichen in Deutschland. Ergebnisse des bundesweiten Kinder- und Jugendgesundheitssurveys (KiGGS). Bundesgesundheitsblatt Gesundheitsforschung Gesundheitsschutz.

[B5] Kromeyer-Hauschild K, Wabitsch M, Kunze D, Geller F, Geiß H, Hesse V, von Hippel A, Jaeger U, Johnsen D, Korte W (2001). Perzentile für den Body-Mass-Index für das Kindes- und Jugendalter unter Heranziehung verschiedener deutscher Stichproben. Monatsschrift für Kinderheilkunde.

[B6] Max Rubner-Institut. Bundesforschungsinstitut für Ernährung und Lebensmittel (2008). Nationale Verzehrs Studie II – Ergebnisbericht Teil 1. Die bundesweite Befragung zur Ernährung von Jugendlichen und Erwachsenen.

[B7] Deutsche Gesellschaft für Ernährung, Österreichische Gesellschaft für Ernährung, Schweizerische Gesellschaft für Ernährungsforschung, Schweizerische Vereinigung für Ernährung (2001). Referenzwerte für die Nährstoffzufuhr.

[B8] Jones G, Richardson M (2007). An objective examination of consumer perception of nutrition information based on healthiness ratings and eye movements. Public Health Nutr.

[B9] World Health Organization (2002). Diet, nutrition and the prevention of chronic diseases. Report of a Joint WHO/FAO Expert Consultation. Technical Report Series 916.

[B10] Baltas G (2001). Nutrition labelling: issues and policies. Eur J Mark.

[B11] Cowburn G, Stockley L (2005). Consumer understanding and use of nutrition labelling: a systematic review. Public Health Nutr.

[B12] Vijwanathan M, Hastak M (2002). The role of summary information in facilitating consumers' comprehension of nutrition information. J Public Policy Mark.

[B13] Pudel V, Spirik J, Westenhöfer J (1996). Zum Informationsnutzen der Lebensmittelkennzeichnung. Informationsnutzen der Lebensmittelkennzeichnung für deutsche Konsumenten als Entscheidungshilfe bei der Lebensmittelauswahl. Ernährungsbericht 1996.

[B14] Drichoutis AC, Lazaridis P, Nayga RM Consumers' use of nutritional labels: a review of research studies and issues. http://www.amsreview.org/articles/drichoutis09-2006.pdf.

[B15] European Comission Council Directive 90/496/EEC of 24 September 1990 on nutrition labelling rules of foodstuffs. http://europa.eu/scadplus/leg/en/lvb/l21092.htm.

[B16] European Commission (2006). CORRIGENDA. Corrigendum to Regulation (EC) No 1924/2006 of the European Parliament and of the Council of 20 December 2006 on nutrition and health claims made on foods. Official Journal of the European Union.

[B17] Navigator (2007). Front of Pack Signpost Labelling. Exploratory Research. Report COI 280040 1095 JS. April 2007.

[B18] Food Standards Agency Food Standards Agency. Signpost labelling research. http://www.food.gov.uk/foodlabelling/signposting/siognpostlabelresearch/.

[B19] Boville C Claire Boville: Making Healthier Choices Easier-Power Point Presentation at the Federal Ministry of Food, Agriculture and Consumer Protection. http://www.bmelv.de/cae/servlet/contentblob/379366/publicationFile/22055/Praesentation_UK.pdf.

[B20] EUFIC EUFIC Press Release from 25/09/2008: One in four UK consumers look for nutrition information on food labels. http://www.eufic.org/jpage/en/page/PRESS/fftid/Consumer-Insights-UK-results/.

[B21] Scott V, Worsley AF (1994). Ticks, claims, tables and food groups: a comparison for nutrition labelling. Health Promot Int.

[B22] Young L, Swinburn B (2002). Impact of the Pick the Tick food information programme on the salt content of food in New Zealand. Health Promot Int.

[B23] PepsiCo It's the symbol that stands for bette choices. http://www.smartspot.com/about.

[B24] Brink J-W van den, Ministry of Health Welfare and Sport Netherlands van den Brink: Healthy Choice Logo. Dutch approach to signposting. Power Point Presentation at the Federal Ministry of Food, Agriculture and Consumer Protection. http://www.bmelv.de/cae/servlet/contentblob/379368/publicationFile/22053/Praesentation_NL.pdf.

[B25] Grunert K, Wills J (2007). A review of European research on consumer response to nutrition information on food labels. Journal of Public Health.

[B26] Feunekes GIJ, Gortemaker IA, Willems AA, Lion R, Kommer M van den (2008). Front-of-pack nutrition labelling: Testing effectiveness of different nutrition labelling formats front-of-pack in four European countries. Appetite.

[B27] Souci SW, Fachmann W, Kraut H (1989). Die Zusammensetzung der Lebensmittel Nährwert-Tabellen 1989/90.

[B28] Souci SW, Fachmann W, Kraut H (1994). Die Zusammensetzung der Lebensmittel Nährwert-Tabellen.

[B29] Fröleke H (2005). Kleine Nährwerttabelle.

[B30] Erhard J (2005). EBISpro für Windows, Version 7.0.

[B31] Food Standards Agency (2007). Front-of-pack Traffic light signpost labelling Technical Guidance.

[B32] The European Food Information Council (2007). Making sense of Guideline Daily Amounts. FOOD TODAY.

[B33] Max Rubner-Institut. Bundesforschungsinstitut für Ernährung und Lebensmittel Nationale Verzehrs Studie II – Ergebnisbericht Teil 2. Die bundesweite Befragung zur Ernährung von Jugendlichen und Erwachsenen. http://www.bmelv.de/cae/servlet/contentblob/378664/publicationFile/25918/NVS_ErgebnisberichtTeil2.pdf.

[B34] Synovate (2005). Qualitative Signpost Labelling. Refinement Research.

[B35] Grunert KG, Wills JM Pan-European consumer research on in-store behaviour, understanding and use of nutrition information on food labels, and nutrition knowledge. http://www.eufic.org/upl/1/default/doc/EUFIC%20pan-European%20results-full%20presentation_FINAL.pdf.

[B36] Kardes FR, Posavac SS, Cronley ML (2004). Consumer Inference: A Review of Processes, Bases, and Judgment Contexts. J Consum Psychol.

[B37] Satia JA, Galanko JA, Neuhouser ML (2005). Food nutrition label use is associated with demographic, behavioral, and psychosocial factors and dietary intake among African Americans in North Carolina. J Am Diet Assoc.

[B38] Neuhouser ML, Kristal AR, Patterson RE (1999). Use of food nutrition labels is associated with lower fat intake. J Am Diet Assoc.

[B39] Kreuter MW, Brennan LK, Scharff DP, Lukwago SN (1997). Do nutrition label readers eat healthier diets? Behavioral correlates of adults' use of food labels. Am J Prev Med.

[B40] Lin CT, Lee JY, Yen ST (2004). Do dietary intakes affect search for nutrient information on food labels?. Soc Sci Med.

[B41] Misra R (2007). Knowledge, attitudes, and label use among college students. J Am Diet Assoc.

