# Supplementary material for: Impact of different food label formats on healthiness evaluation and food choice of consumers: a randomized-controlled study
Source: BMC Public Health. 2009 Jun 12;9:184. doi: 10.1186/1471-2458-9-184 (PMC2702386; doi:10.1186/1471-2458-9-184)
Supplement: Additional file 1 — Percentage of correct choices in the pair-wise comparison task 1. Food pairs used in the pair wise comparison task 1 (the less healthy food in brackets) and percentage of correct choices within each experimental condition (signpost format). The highest percentages are printed bold. [file 1471-2458-9-184-S1.pdf]

Additional file 1:

Food pairs used in the pair wise comparison task 1 (the less healthy food in brackets) and percentage of correct choices within each experimental condition (signpost format). The highest percentages are printed bold.

Compared Food Products		No label n = 84	Tick label n = 84	Traffic light n = 84	GDA n = 84	CGDA n = 84	Total n = 420	Sign.
Orange juice	(Orange nectar)	88.1%	89.3%	**96.4%**	**96.4%**	95.2%	93.1%	-
(Mineral water with fruit flavour) [1]	Mineral water with fruit flavour[1]	50.0%	76.2%	**90.5%**	89.3%	88.1%	78.8%	***
(Apple juice)	Water with apple flavour[1]	40.5%	59.5%	**85.7%**	77.4%	71.4%	66.9%	***
Margarine, 50% reduced-fat	(Margarine with Yoghurt)	33.3%	47.6%	58.3%	**64.3%**	54.8%	51.7%	**
(Salad dressing, French)	Salad dressing, Yoghurt	83.3%	91.7%	**100%**	97.6%	92.9%	93.1%	***
(Butter)	Margarine	72.6%	83.3%	**88.1%**	69.0%	67.9%	76.2%	**
Pasta Sauce with herbs	(Pasta Sauce, Ricotta)	75.0%	82.1%	**97.6%**	91.7%	90.5%	87.4%	***
(Salami, pork)	Salami, poultry	90.5%	88.1%	**98.8%**	**98.8%**	92.9%	93.8%	**
(Ham, rolled filet)	Ham, backside	48.8%	45.2%	**56.0%**	42.9%	54.8%	49.5%	-
Turkey breast	(crumbed chicken fillet) [1]	100%	96.4%	95.2%	**97.6%**	90.5%	96.0%	*
(Ground pork)	Ground beef	76.2%	84.5%	**97.6%**	91.7%	90.5%	88.1%	***
(Tuna in oil)	Pickled herring	72.6%	**75.0%**	61.9%	22.6%	47.6%	56.0%	***
Fried Fishfingers	(Fish filet a la bordelaise)	48.8%	36.9%	**64.3%**	52.4%	**64.3%**	53.3%	**
(Milk , 3.5 %fat)	Reduced-fat Milk, 1.5 %	89.2%	92.9%	**95.2%**	84.5%	89.3%	90.2%	-

Compared Food Products		No label<br>n = 84	Tick label<br>n = 84	Traffic light<br>n = 84	GDA<br>n = 84	CGDA<br>n = 84	Total<br>n = 420	Sign.
Fruit flavoured yoghurt, reduced-fat[1]	(Fruit flavoured yoghurt) [1]	73.5%	82.1%	**84.5%**	79.8%	**84.5%**	80.9%	-
(Full fat yoghurt) [1]	Fruit flavoured yoghurt	78.3%	76.2%	97.6%	**98.8%**	91.7%	88.5%	***
Whipping cream, reduced-fat[1]	(Whipping cream) [1]	74.7%	86.9%	**94.0%**	90.5%	90.5%	87.4%	**
(Crème fraîche)	Crème légère	75.9%	90.5%	**96.4%**	89.3%	91.7%	88.8%	**
Crème finesse	(Crème légère)	48.2%	52.4%	**95.2%**	91.7%	81.0%	73.7%	***
Camembert, light	(Camembert)	85.5%	90.5%	**98.8%**	86.9%	92.9%	90.9%	*
Cheese, reduced-fat[1]	(Cheese, Gouda)	80.7%	89.3%	**97.6%**	89.3%	92.9%	90.0%	**
Granola with fruit	(Granola Crunchy)	85.5%	83.3%	**100%**	97.6%	97.6%	92.8%	***
Whole grain bread	(Bread rye and wheat)	88.0%	**88.1%**	81.0%	63.1%	79.8%	80.0%	***
Whole grain toast	(Sandwich toast)	97.0%	**100%**	96.4%	89.3%	94.0%	95.5%	*
(Frozen Vegetables, buttered)	Frozen Vegetables[1]	90.4%	95.2%	**98.8%**	95.2%	95.2%	95.0%	-
Fruit spread	(Jam)	68.7%	58.3%	**94.0%**	92.9%	88.1%	80.4%	***
Pretzel sticks	(roasted peanuts)	91.6%	**96.0%**	95.2%	92.9%	95.2%	94.3%	-
Milk chocolate	(Dark chocolate)	26.5%	15.5%	**59.5%**	46.4%	42.9%	38.2%	***

Sign.: Significance of Chi-Square-Test: - not significant; * $p < 0.05$; ** $p < 0.01$; *** $p < 0.001$

[1] for the participants the German trade name, but not the brand was visible
