# Supplementary material for: Impact of different food label formats on healthiness evaluation and food choice of consumers: a randomized-controlled study
Source: BMC Public Health. 2009 Jun 12;9:184. doi: 10.1186/1471-2458-9-184 (PMC2702386; doi:10.1186/1471-2458-9-184)
Supplement: Additional file 2 — Envisaged daily consumption of energy and nutrients (gram and energy percent) in the virtual grocery task (task 2). The data provided represent the mean ± standard deviation of the envisaged daily consumption of energy and nutrients in the virtual grocery task. [file 1471-2458-9-184-S2.pdf]

Additional file 2:

Envisaged daily consumption of energy and nutrients (gram and energy percent) in the virtual grocery task (task 2)

Energy / nutrient	Gender	Unit	experimental condition: No label	Tick label	Traffic light	GDA	CGDA	Total
Energy	male	kcal	3161.4 ± 1555.3	2901.0 ± 1193.8	2914.5 ± 923	2780.0 ± 842	2980.6 ± 1375.5	2945.0 ± 1199.0
	female	kcal	2091.0 ± 695.1	2037.6 ± 736.4	2074.8 ± 873.3	1912.8 ± 652.8	2085.9 ± 945.2	2039.3 ± 778.3
	total	kcal	2537.9 ± 1245.9	2448.5 ± 1067.1	2474.6 ± 986.8	2294.7 ± 855.1	2543.9 ± 1261.1	2459.8 ± 1092.7
fat	male	g	141.7 ± 96.1	135.5 ± 78.0	114.2 ± 55.2	121.6 ± 53.7	133.8 ± 79.5	129.2 ± 73.9
		%	38.3 ± 7.9	40.0 ± 9.0	34.3 ± 9.7	38.8 ± 10.4	38.7 ± 10.2	38.0 ± 9.6
	female	g	77.8 ± 43.5	81.0 ± 42.7	82.0 ± 48.1	73.2 ± 31.9	83.3 ± 51.4	79.3 ± 43.5
		%	32.1 ± 10.1	34.5 ± 7.7	33.6 ± 8.8	34.1 ± 8.4	34.6 ± 8.7	33.7 ± 8.8
	total	g	104.4 ± 76.7	107.0 ± 67.5	97.3 ± 53.8	94.5 ± 49	109.2 ± 71.5	102.5 ± 64.5
		%	34.7 ± 9.7	37.1 ± 8.7	33.9 ± 9.2	36.2 ± 9.6	36.7 ± 9.7	35.7 ± 9.4
saturated fat	male	g	53.6 ± 33.9	53.1 ± 30.1	44.1 ± 23.2	45.0 ± 21.1	51.2 ± 33.0	49.4 ± 28.7
		%	14.8 ± 4.2	15.8 ± 4.7	13.3 ± 5.1	14.3 ± 4.5	14.8 ± 6.1	14.6 ± 5.0
	female	g	30.2 ± 17.0	30.5 ± 16.1	28.7 ± 16.6	26.9 ± 12.4	32.9 ± 21.9	29.7 ± 16.9
		%	12.6 ± 4.9	13.1 ± 3.5	11.9 ± 4.0	12.7 ± 4.2	13.6 ± 5.3	12.8 ± 4.4
	total	g	49.9 ± 27.8	41.2 ± 26.3	36.0 ± 21.3	34.9 ± 19.0	42.3 ± 29.4	38.9 ± 25.1
		%	13.5 ± 4.8	14.3 ± 4.3	12.6 ± 4.6	13.4 ± 4.4	14.2 ± 5.7	13.6 ± 4.8

Envisaged daily consumption of energy and nutrients (gram and energy percent) in the virtual grocery task (task 2) (continued)

Energy / nu-			experimental condition					
trient	Gender	Unit	No label	Tick label	Traffic light	GDA	CGDA	Total
sugar	male	g	161.3 ± 77.4	137.5 ± 67.4	162.2 ± 67.0	149.2 ± 71.0	143.5 ± 85.1	150.4 ± 73.9
		%	21.6 ± 6.8	20.0 ± 8.2	23.2 ± 7.8	22.3 ± 8.5	20.0 ± 7.9	21.4 ± 7.9
	female	g	129.7 ± 44.2	120.5 ± 61.8	112.5 ± 56.8	115.6 ± 55.5	121.5 ± 53.2	120.1 ± 54.3
		%	26.0 ± 7.2	24.7 ± 9.7	22.6 ± 7.9	24.5 ± 6.9	24.8 ± 7.2	24.6 ± 7.8
	total	g	142.8 ± 61.9	128.6 ± 64.7	136.2 ± 66.3	130.4 ± 64.6	132.8 ± 71.7	134.2 ± 65.8
		%	24.2 ± 7.3	22.4 ± 9.2	22.9 ± 7.8	23.5 ± 7.7	22.4 ± 7.9	23.1 ± 8
sodium	male	g	6.3 ± 4.0	5.9 ± 3.3	5.3 ± 2.6	4.9 ± 2.3	6.1 ± 3.3	5.7 ± 3.2
	female	g	3.6 ± 1.4	3.6 ± 1.4	3.9 ± 1.7	3.2 ± 1.2	3.5 ± 2.3	3.5 ± 1.6
	total	g	4.7 ± 3.1	4.7 ± 2.7	4.5 ± 2.3	3.9 ± 2.0	4.8 ± 3.1	4.5 ± 2.7
protein	male	g	116.6 ± 62.9	115.3 ± 46.8	118.0 ± 51.4	101.5 ± 41.2	120.3 ± 54.7	114.6 ± 51.7
		%	14.7 ± 2.8	16.3 ± 3.8	16.4 ± 4.9	14.5 ± 3.0	16.7 ± 4.9	15.8 ± 4.1
	female	g	77.4 ± 27.9	76.8 ± 29.6	78.3 ± 31.7	74.6 ± 26.3	79.1 ± 40.3	77.2 ± 31.0
		%	14.9 ± 3.2	15.3 ± 3.8	15.6 ± 3.3	16.0 ± 4.3	15.3 ± 3.5	15.4 ± 3.6
	total	g	93.7 ± 49.5	95.2 ± 43.1	97.2 ± 46.5	86.4 ± 36.1	100.1 ± 52.2	94.5 ± 45.8
		%	14.8 ± 3	15.8 ± 3.8	16.0 ± 4.1	15.4 ± 3.9	16.0 ± 4.3	15.6 ± 3.9

Envisaged daily consumption of energy and nutrients (gram and energy percent) in the virtual grocery task (task 2) (continued)

Energy / nutrient	Gender	Unit	experimental condition					
			No label	Tick label	Traffic light	GDA	CGDA	Total
carbohydrate	male	g	344.3 ± 130.6	296.7 ± 105.4	343.0 ± 117.8	308.0 ± 112.5	313.1 ± 144.3	320.5 ± 123.4
		%	45.4 ± 8.0	42.7 ± 9.7	47.7 ± 10.6	45.0 ± 9.8	43.1 ± 9.8	44.7 ± 9.7
	female	g	262.5 ± 81.0	242.6 ± 84.1	249.4 ± 93.1	231.2 ± 91.3	246.9 ± 101.2	246.7 ± 89.9
		%	51.5 ± 10.0	48.6 ± 8.8	49.6 ± 8.1	48.1 ± 8.9	48.5 ± 9.1	49.3 ± 9.0
	total	g	296.6 ± 111.5	268.3 ± 98.1	294.0 ± 115.0	265.1 ± 107.6	280.8 ± 128.8	281.0 ± 112.8
		%	49.0 ± 9.6	45.8 ± 9.6	48.7 ± 9.4	46.7 ± 9.4	45.7 ± 9.8	47.2 ± 9.6
energy density	male	kcal/g	0.8 ± 0.2	0.8 ± 0.2	0.7 ±0.2	0.7 ±0.2	0.8 ±0.3	0.8 ±0.2
	female	kcal/g	0.6 ± 0.2	0.6 ± 0.2	0.6 ± 0.3	0.6 ±0.2	0.6 ±0.2	0.6 ±0.2
	total	kcal/g	0.7 ± 0.2	0.7 ± 0.2	0.7 ± 0.2	0.7 ±0.3	0.7 ±0.2	0.7 ±0.2

%: per cent of total energy intake
