# Supplementary material for: Impact of different food label formats on healthiness evaluation and food choice of consumers: a randomized-controlled study
Source: BMC Public Health. 2009 Jun 12;9:184. doi: 10.1186/1471-2458-9-184 (PMC2702386; doi:10.1186/1471-2458-9-184)
Supplement: Additional file 3 — Total envisaged daily consumption compared with the results of the German National Nutrition Survey II (NVS II). The data provided represent the mean ± standard deviation and the median of the envisaged daily consumption of energy and nutrients from the total sample (all experimental conditions combined) in the virtual grocery task and a comparison with the mean and median of intakes in the German National Nutrition Survey II (NVS II) [33]. [file 1471-2458-9-184-S3.pdf]

Additional file 3:

Total envisaged daily consumption compared with the results of the German National Nutrition Survey II (NVS II)[1] (Max Rubner-Institut. Bundesforschungsinstitut für Ernährung und Lebensmittel, 2008)

Energy/ nutrient	Gender	Unit	Food Labelling study (total)		NVS II	
			Mean ± SD	Median	Mean	Median
Energy	male	kcal	2945.0 ± 1199.0	2759	2571	2413
	female	kcal	2039.3 ± 778.3	1900	1915	1833
fat	male	g	129.2 ± 73.9	113.3	102.0	92.5
		%	38.0 ± 9.6	38.8	35.8	35.8
	female	g	79.3 ± 43.5	71.8	73.8	68.4
		%	33.7 ± 8.8	34.1	34.8	34.7
sodium	male	g	5.7 ± 3.2	4.7	3.5	3.2
	female	g	3.5 ± 1.6	3.3	2.5	2.4
protein	male	g	114.6 ± 51.7	99.7	90.8	85.2
	female	g	77.2 ± 31.0	70.4	66.7	64.3
carbohydrates	male	g	320.5 ± 123.4	307.1	289.2	270.2
		%	44.7 ± 9.7	44.6	45.5	45.3
	female	g	246.7 ± 89.9	238.2	231.0	220.2
		%	49.3 ± 9.0	48.5	48.9	48.9

[1] only parameters available in the published report from NVS II are presented
